# USING OSJ PUBLISHING AND TEAM PEER REVIEWS TO EXPAND SCHOLARLY WORKS FROM A DIVERSIFIED AND INCLUSIVE PERSPECTIVE

**DOI:** 10.1093/geroni/igz038.1317

**Published:** 2019-11-08

**Authors:** Pamela P Brown

**Affiliations:** Albany State University, Albany, Georgia, United States

## Abstract

While Minority Serving Institutions (MSI) are often more teaching than research focused, promotion and tenure heavily rest on the success of being published. It is challenging to publish with high teaching loads and limited research time. A viable alternative is to publish the scholarship of teaching/learning work conducted within MSI classrooms. Open Source Journals (OSJ) assists with the publication needs of MSI faculty and can bring a more diversified andragogic practice out to the public sphere. It also allows for the publishing of more peer-reviewed journals, thus allowing for a greater expansion of diversity in the editorial process. This presentation will focus on a newly created OSJ, which focuses on MSI faculty and innovative teaching. A discussion of a team peer review process is included which focuses on the ability to be inclusive in publishing scholarly works from MSI faculty and those who work with historically underserved student populations.

